# Characterization of monocarboxylate transporters (MCTs) expression in soft tissue sarcomas: distinct prognostic impact of MCT1 sub-cellular localization

**DOI:** 10.1186/1479-5876-12-118

**Published:** 2014-05-09

**Authors:** Céline Pinheiro, Valter Penna, Filipa Morais-Santos, Lucas F Abrahão-Machado, Guilherme Ribeiro, Emílio C Curcelli, Marcus V Olivieri, Sandra Morini, Isabel Valença, Daniela Ribeiro, Fernando C Schmitt, Rui M Reis, Fátima Baltazar

**Affiliations:** 1 Life and Health Sciences Research Institute, School of Health Sciences, University of Minho, 4710-057 Braga, Portugal; 2 ICVS/3B’s - PT Government Associate Laboratory, Guimarães, Braga, Portugal; 3 Barretos School of Health Sciences, Dr. Paulo Prata - FACISB, São Paulo, Barretos, Brazil; 4 Molecular Oncology Research Center, Barretos Cancer Hospital, Pio XII Foundation, São Paulo, Barretos, Brazil; 5 Department of Orthopedics, Barretos Cancer Hospital, Pio XII Foundation, São Paulo, Barretos, Brazil; 6 Department of Pathology, Barretos Cancer Hospital, Pio XII Foundation, São Paulo, Barretos, Brazil; 7 Medical Faculty, UNESP, Botucatu, São Paulo, Brazil; 8 Centre for Cell Biology and Department of Biology, University of Aveiro, Aveiro, Portugal; 9 Medical Faculty, University of Porto, Porto, Portugal; 10 IPATIMUP – Institute of Molecular Pathology and Immunology of University of Porto, Porto, Portugal; 11 Department of Laboratory Medicine & Pathobiology, Faculty of Medicine, University of Toronto, Toronto, Canada

**Keywords:** Monocarboxylate transporters, CD147/EMMPRIN, Soft tissue sarcoma, Prognosis

## Abstract

**Background:**

Soft tissue sarcomas (STSs) are a group of neoplasms, which, despite current therapeutic advances, still confer a poor outcome to half of the patients. As other solid tumors, STSs exhibit high glucose consumption rates, associated with worse prognosis and therapeutic response. As highly glycolytic tumors, we hypothesized that sarcomas should present an increased expression of lactate transporters (MCTs).

**Methods:**

Immunohistochemical expression of MCT1, MCT2, MCT4 and CD147 was assessed in a series of 86 STSs and the expression profiles were associated with patients’ clinical-pathological parameters.

**Results:**

MCT1, MCT4 and CD147 were mainly observed in the plasma membrane of cancer cells (around 60% for MCTs and 40% for CD147), while MCT2 was conspicuously found in the cytoplasm (94.2%). Importantly, we observed MCT1 nuclear expression (32.6%). MCT1 and MCT4, alone or co-expressed with CD147 in the plasma membrane, were associated with poor prognostic variables including high tumor grade, disease progression and shorter overall survival. Conversely, we found MCT1 nuclear expression to be associated with low grade tumors and longer overall survival.

**Conclusions:**

The present work represents the first report of MCTs characterization in STSs. We showed the original finding of MCT1 expression in the nucleus. Importantly, opposite biological roles should be behind the dual sub-cellular localization of MCT1, as plasma membrane expression of MCT1 is associated with worse patients’ prognosis, while nuclear expression is associated with better prognosis.

## Background

Soft tissue sarcomas (STSs) are an extremely heterogeneous group of rare tumors that arise predominantly from the embryonic mesoderm [1]. STSs can occur in any soft tissue in the body, and may have different etiologies, including external radiation therapy and occupational exposure to certain chemicals such as herbicides [1,2]. Despite some therapeutic improvements, metastasis and death remain a significant problem in half of STS patients, who present advanced disease [1]. Recent advances on the knowledge related to the oncogenic mechanisms underlying sarcomagenesis will hopefully translate into more effective therapies [1,2].

It has been shown that most sarcomas exhibit a strong glycolytic phenotype and this phenomenon supplies the rationale for positron emission tomography imaging of STS using ^18^F-fluorodeoxyglucose (FDG-PET) [3]. FDG-PET measurements in STS allow primary and recurrent detection of intermediate and high-grade tumors [4], being associated with high histological grade [3-5], cellularity, proliferative activity, MIB labeling index, and p53 overexpression [5]. Additionally, FDG-PET is proposed as a modality to monitor treatment response in high-grade STS patients [3,6,7], as quantitative FDG-PET is significantly more accurate than size-based criteria at assessing STS response to neoadjuvant therapy [6,7].

The specific changes that occur in tumor microenvironment have been recently identified as key components in carcinogenesis. In fact, the reprogramming of energy metabolism, with special focus on the Warburg phenomenon, i.e., the preference for the glycolytic phenotype even in the presence of oxygen, has been recently included as a “new” hallmark of cancer [8]. Since the hyper-glycolytic phenotype will generate high amounts of lactate inside cancer cells, monocarboxylate transporters (MCTs) play an important role in the extrusion of lactate, contributing to the maintenance of the intracellular pH of tumor cells. MCTs are transmembrane proteins, which are encoded by the family of genes *SLC16A*, which presently include 14 members. However, only the first four members, MCT1-4, are able to transport lactate and other monocarboxylates across membranes, coupled with a proton [9]. MCT1 has a ubiquitous distribution in human tissues, being involved in both uptake and efflux of monocarboxylates from cells, and is considered an intermediate affinity isoform. MCT2 is a high affinity transporter, being adapted to the uptake of monocarboxylates into cells, and is mostly found in tissues that use lactate as a respiratory fuel. MCT3 has a very restricted distribution, having been identified in the retinal pigment and choroid plexus epithelia. MCT4 is known as a low affinity transporter and has been observed particularly in highly glycolytic tissues, where is essentially responsible for lactate efflux. MCT1 and MCT4 are the best studied isoforms in cancer, as being responsible for lactate transport across the plasma membrane [9].

However, the role of MCTs in solid tumors is still far from being fully characterized [10]. Although pointed out as potential therapeutic targets [11-15], more information on MCTs’ expression in human solid tumors is needed to further translate knowledge into the clinic context. Some information about the expression and clinical-pathological significance of MCTs is provided for various human tumors [10] like tumors from brain [16], colon [17,18], breast [19], uterine cervix [20], prostate [21] and lung [22], and the vast majority of studies are focused on tumors of epithelial lineage, but similar studies are lacking for other types of tumors. In sarcomas, MCT1 expression was found in the precrystalline cytoplasmic granules of 7 out of 10 alveolar soft part sarcomas [23], while MCT1, MCT2 and MCT4 mRNAs were found in the human rhabdomyosarcoma cell line RD [24-26]. Therefore, studies on MCT expression and clinical-pathological significance in sarcomas may contribute to the knowledge on the biology of this tumor.

Therefore, the aim of this work was to assess the expression of MCT1, MCT2 and MCT4, and MCT1/4 chaperone CD147, in a series of sarcomas, and evaluate their clinical-pathological significance.

## Methods

### Case selection

Eighty-six samples of soft tissue sarcomas were retrieved from the Pathology archives of Barretos Cancer Hospital, Barretos, São Paulo, Brazil. Relevant clinical-pathological data available included patient’s age, gender and race, tumor localization, diagnostic and grade (according to the French Federation of Cancer Centers Sarcoma Group (FNCLCC) [27]), cell lineage, presence of pleomorphic cells, disease progression, disease recurrence and presence of metastasis, as specified in Table 1. Moreover, follow-up information was available for 84 patients and overall survival was defined as the time between the date of first consultation and the date of last information or patient death. This study was approved by the local Ethics Committee (Barretos Cancer Hospital; no. 331/2010).

**Table 1:** Clinical-pathological data of sarcomas’ patients

**Variable**	**n**	**%**
**Age**		
>51	**48**	56.5
≤51	**37**	43.5
**Gender**		
Female	**33**	38.8
Male	**52**	61.2
**Race**		
Caucasian	**63**	74.1
Not Caucasian	**22**	25.9
**Local**		
Lower extremities	**63**	73.3
Upper extremities	**23**	26.7
**Diagnostic**		
Pleomorphic leiomyosarcoma	**17**	19.8
Myxoid/round cells liposarcoma	**14**	16.3
High grade undifferentiated pleomorphic sarcoma	**12**	14.0
Monophasic fibrous synovial sarcoma	**9**	10.5
Myxofibrosarcoma	**7**	8.1
Malignant peripheral nerve sheath tumor	**7**	8.1
Low grade fibromyxoid sarcoma	**3**	3.5
Poorly differentiated synovial sarcoma	**2**	2.3
High grade myofibroblastic sarcoma	**2**	2.3
Clear cell sarcoma	**1**	1.2
Biphasic synovial sarcoma	**1**	1.2
Fibrosarcoma	**1**	1.2
Angiomatoid histiocytoma	**1**	1.2
Infantile fibrosarcoma	**1**	1.2
Well differentiated liposarcoma	**1**	1.2
Undifferentiated liposarcoma	**1**	1.2
Pleomorphic liposarcoma	**1**	1.2
Acral myxoinflammatory fibroblastic sarcoma	**1**	1.2
Low grade myofibroblastic sarcoma	**1**	1.2
Giant-cell-rich high grade undifferentiated pleomorphic sarcoma	**1**	1.2
Malignant solitary fibrous tumor	**1**	1.2
Alveolar sarcoma	**1**	1.2
**Cell lineage**		
Fibroblastic/myofibroblastic	**28**	32.5
Smooth muscle	**17**	19.8
Lipogenic	**17**	19.8
Peripheral nerve	**7**	8.1
Miscellaneous	**17**	19.8
**Cellular pleomorphism**		
Absence of pleomorphic cells	**54**	62.8
Presence of pleomorphic cells	**32**	37.2
**Grade**		
Low grade (I)	**30**	32.2
High grade (II e III)	**63**	67.7
**Disease progression**		
No	**25**	30.9
Yes	**56**	69.1
**Disease recurrence**		
Absent	**48**	57.1
Present	**36**	42.9
**Metastasis**		
Absent	**44**	52.4
Present	**40**	47.6

Significant values are shown in bold.

### Immunohistochemistry

#### MCT and CD147 detection

MCT1 immunohistochemistry was performed according to the avidin-biotin-peroxidase complex method (R.T.U. VECTASTAIN Elite ABC Kit (Universal), Vector Laboratories, Burlingame, CA), with primary antibody for MCT1 (AB3538P, Chemicon International, Temecula, CA) diluted 1:200, as previously described [17]. Immunohistochemistry for MCT2, MCT4 and CD147 was performed according to the streptavidin-biotin-peroxidase complex principle (Ultravision Detection System Anti-polyvalent, HRP, Lab Vision Corporation, Fremont, CA), using primary antibodies raised against MCT2 (sc-50322, Santa Cruz Biotechnology, Santa Cruz, CA), MCT4 (sc-50329, Santa Cruz Biotechnology, Santa Cruz, CA), and CD147 (18–7344, ZYMED Laboratories Inc., South San Francisco, CA), diluted 1:100, 1:500 and 1:750, respectively, as previously described [28]. Negative controls were performed by the use of appropriate serum controls for the primary antibodies (N1699, Dako, Carpinteria, CA). Colon carcinoma tissue was used as positive control for MCT1, MCT4 and CD147 while kidney was used for MCT2. Tissue sections were counterstained with hematoxylin and permanently mounted.

#### Immunohistochemical evaluation

Sections were scored semi-quantitatively for plasma membrane expression as follows: 0: 0% of immunoreactive cells; 1: <5% of immunoreactive cells; 2: 5-50% of immunoreactive cells; and 3: >50% of immunoreactive cells. Also, intensity of staining was scored semi-qualitatively as follows: 0: negative; 1: weak; 2: intermediate; and 3: strong. The final score was defined as the sum of both parameters (extension and intensity), and grouped as negative (score 0 and 2) and positive (score 3–6), as previously described [17]. Protein expression in other cellular localizations was also assessed. Two independent pathologists (LM and FCS) performed immunohistochemical evaluation blindly and discordant results were discussed in a double-head microscope in order to determine the final score.

### Immunofluorescence

MLS-1765 cells, a myxoid liposarcoma cell line provided by Dr. Pierre Åman (Lundberg Laboratory for Cancer Research, Department of Pathology, Sahlgrenska Academy at Göteborg University, Göteborg, Sweden), were subjected to immunofluorescence to determine the subcellular localization of MCT1, MCT4 and CD147. Cells were seeded on glass cover slips and fixed after 24 h with 4% paraformaldehyde in PBS, pH 7.4, during 20 min. Afterwards, cells were permeabilized with 0.2% Triton X-100 for 10 min, blocked with 1% BSA solution for further 10 min and incubated with the same primary antibodies used for immunohistochemistry, for 1 h. Upon 1 h incubation with the secondary antibody (Fluorophore Alexa 488, Invitrogen, Life Technologies, Grand Island, NY, USA), cells were stained with Hoechst 33342 and mounted on slides using Mowiol 4–88 containing n-propylgallate. Between each step, cells were extensively rinsed 3 times with PBS, pH 7.4. Images were obtained using a Zeiss LSM 510 Meta Confocal setup (Carl Zeiss, Oberkochen, Germany) equipped with a plan-Apochromat 100×/1.4 oil objective.

### Cell fractionation and Western blot

Nuclear and cytosolic proteins were extracted from MLS-1765 cells, with a nuclear extraction kit (NXTRACT, Sigma-Aldrich) according to the manufacturer’s instructions.

Protein quantification was performed according to the Bio-Rad Dc Protein Assay (500–0113, Bio Rad) and Western blot was performed as previously described [16]. Briefly, after incubation with the primary polyclonal antibodies rabbit anti-MCT1 (1:200 dilution; AB3538P; Chemicon International), rabbit anti-Histone H3 (1:1000 dilution; ab1791, Abcam), membranes were incubated with the respective secondary antibody coupled to horseradish peroxidase (SantaCruz Biotechnology). The bound antibodies were visualized by chemiluminescence (Supersignal West Femto kit; Pierce).

### Statistical analysis

Data were stored and analyzed using the IBM SPSS statistical software (version 19, IBM Company, Armonk, NY). All comparisons were examined for statistical significance using Pearson’s chi-square (χ^2^) test and Fisher’s exact test (when n < 5), being threshold for significance *p* values <0.05. Overall survival curves were estimated by the method of Kaplan-Meier and data compared using the log rank test. Cases lacking one or more of the clinical-pathological variables were not included in the specific statistical analysis.

## Results

### Soft tissue sarcomas show nuclear expression of MCT1

**Figure 1:**
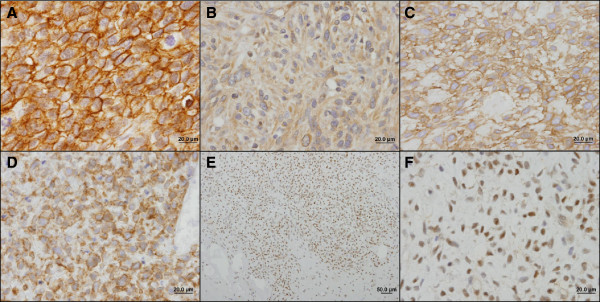
**Representative immunohistochemical reactions for MCT1, MCT2, MCT4 and CD147 in soft tissue sarcomas. (A)** High grade undifferentiated pleomorphic sarcoma (fibroblastic/myofibroblastic cell lineage) showing MCT1 plasma membrane expression; **(B)** Malignant solitary fibrous tumor (miscellaneous cell lineage) showing cytoplasmatic expression of MCT2; **(C)** Malignant peripheral nerve sheath tumor (peripheral nerve cell lineage) showing MCT4 expression in the plasma membrane; **(D)** Myxoid/round cells liposarcoma (lipogenic cell lineage) showing CD147 in the plasma membrane; **(E and F)** Myxoid/round cells liposarcoma (lipogenic cell lineage) showing nuclear expression of MCT1. **A**-**D** and **F**: 400× magnification; **E**: 100× magnification.

A series of 86 cases of histologically confirmed soft tissue sarcomas was analyzed for the immunohistochemical expression of the MCT isoforms 1, 2 and 4 as well as MCT1 and 4 chaperone, CD147. Overall, the expression of these proteins was mainly found at the plasma membrane (Figure 1), with the exception of MCT2 (Figure 1B), which was only observed in the plasma membrane in one case. MCT1 plasma membrane expression was depicted in 52 cases (60.5%, Figure 1A), MCT2 expression was found in 81 cases (94.2%), MCT4 was observed in the plasma membrane of 49 cases (57.0%, Figure 1C), while CD147 was found in 38 cases (44.2%, Figure 1D). Importantly, nuclear expression of MCT1 was observed in 28 cases (32.6%, Figure 1E and F). Additionally, a significant association between plasma membrane expression of MCT1/4 and CD147 was found (Table 2).

**Table 2:** Association of CD147 with MCT1 and MCT4 expression in sarcoma samples

	**n**	**CD147 positive (%)**	***p***
**MCT1**			**<0.001**
Negative	**34**	3 (8.8)	
Positive	**52**	36 (67.3)	
**MCT4**			**0.005**
Negative	**37**	10 (27.0)	
Positive	**49**	29 (57.1)	

Significant values are shown in bold.

### Plasma membrane expression of MCT1 and MCT4 is associated with poor prognostic features, while nuclear expression of MCT1 is associated with good prognosis

Regarding the association between the clinical-pathological features and the expression of the proteins herein analyzed, many important associations were found (Tables 3 and 4). Firstly, despite the lack of statistical association, we observed a tendency of MCT1 expression with cell lineage. We found MCT1 expression to be associated with the presence of pleomorphic cells (*p* = 0.034), high grade tumors (*p* < 0.001) and disease progression (*p* = 0.018). Importantly, nuclear expression of MCT1, was associated with cell lineage (*p* < 0.001), being evidently more frequently observed in the lipogenic lineage. Additionally, MCT1 nuclear expression was significantly associated with the absence of pleomorphic cells (*p* = 0.010) and low grade tumors (*p* < 0.001) and showed a tendency to be more frequent in patients without metastasis (*p* = 0.084) and younger patients (*p* = 0.076). Concerning MCT4 expression, similar associations to the ones depicted for MCT1 were found; MCT4 was more frequently expressed in the smooth muscle lineage (*p* < 0.001), was associated with the presence of pleomorphic cells (*p* = 0.009), high grade tumors (*p* = 0.011) and showed a tendency to be associated with disease progression (*p* = 0.060). CD147 expression alone was associated with disease progression (*p* = 0.020) and showed a tendency to be associated with presence of pleomorphic cells (p = 0.083) and high tumor grade (*p* = 0.076), associations that reached significance when CD147 was evaluated in co-expression either with MCT1 or MCT4. As the majority of cases were positive for cytoplasmic expression of MCT2 and only one showed plasma membrane expression, no statistical analysis was performed for this protein.

**Table 3:** Association of MCT expression with the clinical-pathological parameters

***Clinical-pathological data***	***MCT1 (PM)***			***MCT1 (nucleus)***		***MCT4 (PM)***	
	**n**	**Positive (%)**	***p***	**Positive (%)**	***p***	**Positive (%)**	***p***
**Age**			0.153		0.076		0.403
>51	**48**	32 (66.7)		12 (25.0)		29 (60.4)	
≤51	**37**	19 (51.4)		16 (43.2)		19 (51.4)	
**Gender**			0.716		0.174		0.463
Female	**33**	19 (57.6)		8 (24.2)		17 (51.5)	
Male	**52**	32 (61.5)		20 (38.5)		31 (59.6)	
**Race**			0.686		0.896		0.832
Caucasian	**63**	37 (58.7)		21 (33.3)		36 (57.1)	
Not Caucasian	**22**	14 (63.6)		7 (31.8)		12 (54.5)	
**Local**			0.586		0.439		0.154
Lower extremities	**63**	37 (58.7)		22 (34.9)		33 (52.4)	
Upper extremities	**23**	15 (65.2)		6 (26.1)		16 (69.6)	
**Cell lineage**			0.066		**<0.001**		**<0.001**
Fibroblastic/myofibroblastic	**28**	16 (57.1)		9 (32.1)		16 (57.1)	
Smooth muscle	**17**	12 (70.6)		3 (17.6)		13 (76.5)	
Lipogenic	**17**	6 (35.3)		13 (76.5)		2 (11.8)	
Peripheral nerve	**7**	4 (57.1)		2 (28.6)		4 (57.1)	
Miscellaneous	**17**	14 (82.4)		1 (5.9)		14 (82.4)	
**Cellular pleomorphism**			**0.034**		**0.010**		**0.009**
Absence of pleomorphic cells	**54**	28 (51.9)		23 (42.6)		25 (46.3)	
Presence of pleomorphic cells	**32**	24 (75.0)		5 (15.6)		24 (75.0)	
**Grade**			**<0.001**		**<0.001**		**0.011**
Low grade (I)	**19**	4 (21.1)		14 (73.7)		6 (31.6)	
High grade (II e III)	**67**	48 (71.6)		14 (20.9)		43 (64.2)	
**Disease progression**			**0.018**		0.865		0.060
No	**25**	10 (40.0)		8 (32.0)		10 (40.0)	
Yes	**56**	39 (67.2)		20 (33.9)		35 (62.5)	
**Disease recurrence**			0.847		0.640		0.204
Absent	**48**	29 (60.4)		15 (31.2)		24 (50.0)	
Present	**36**	21 (58.3)		13 (36.1)		23 (63.9)	
**Metastasis**			0.156		0.122		0.249
Absent	**44**	23 (52.3)		18 (40.9)		22 (50.0)	
Present	**40**	27 (67.5)		10 (25.0)		25 (62.5)	

*PM*- Plasma membrane.

Significant values are shown in bold.

**Table 4:** Association between CD147 plasma membrane expression, alone or co-expressed with MCT1, and the clinical-pathological parameters

***Clinical-pathological data***	***CD147***			***CD147 + MCT1***		***CD147 + MCT4***	
	**n**	**Positive (%)**	***p***	**Positive (%)**	***p***	**Positive (%)**	***p***
**Age**			0.498		0.583		0.308
>51	**48**	23 (47.9)		21 (43.8)		18 (37.5)	
≤51	**37**	15 (40.5)		14 (37.8)		10 (27.0)	
**Gender**			0.577		0.523		0.680
Female	**33**	16 (48.5)		15 (45.5)		10 (30.3)	
Male	**52**	22 (42.3)		29 (38.5)		19 (34.6)	
**Race**			0.562		0.976		0.511
Caucasian	**63**	27 (42.9)		26 (41.3)		22 (34.9)	
Not Caucasian	**22**	11 (50.0)		9 (40.9)		6 (27.3)	
**Local**			0.936		0.751		0.432
Lower extremities	**63**	28 (44.4)		25 (39.7)		19 (30.2)	
Upper extremities	**23**	10 (43.5)		10 (43.5)		9 (39.1)	
**Cell lineage**			0.878		0.694		0.071
Fibroblastic/myofibroblastic	**28**	13 (46.4)		12 (42.9)		12 (42.9)	
Smooth muscle	**17**	6 (35.3)		6 (35.3)		5 (29.4)	
Lipogenic	**17**	7 (41.2)		5 (29.4)		1 (5.9)	
Peripheral nerve	**7**	3 (42.9)		3 (42.9)		2 (28.6)	
Miscellaneous	**17**	9 (52.9)		9 (52.9)		8 (47.1)	
**Cellular pleomorphism**			0.083		0.071		**0.002**
Absence of pleomorphic cells	**54**	20 (37.0)		18 (33.3)		11 (20.4)	
Presence of pleomorphic cells	**32**	18 (56.2)		17 (53.1)		17 (53.1)	
**Grade**			0.076		**0.016**		**0.026**
Low grade (I)	**19**	5 (26.3)		3 (15.8)		2 (10.5)	
High grade (II e III)	**67**	33 (49.3)		32 (47.8)		26 (38.8)	
**Disease progression**			**0.020**		**0.016**		0.070
No	**25**	6 (24.0)		5 (20.0)		4 (16.0)	
Yes	**56**	29 (51.8)		27 (48.2)		21 (37.5)	
**Disease recurrence**			0.949		0.847		0.840
Absent	**48**	21 (43.8)		19 (39.6)		15 (31.2)	
Present	**36**	16 (44.4)		15 (41.7)		12 (33.3)	
**Metastasis**			0.295		0.090		0.593
Absent	**44**	17 (38.6)		14 (31.8)		13 (29.5)	
Present	**40**	20 (50.0)		20 (50.0)		14 (35.0)	

Significant values are shown in bold.

### Plasma membrane expression of MCT1 and MCT4 is associated with shorter patient overall survival

**Figure 2:**
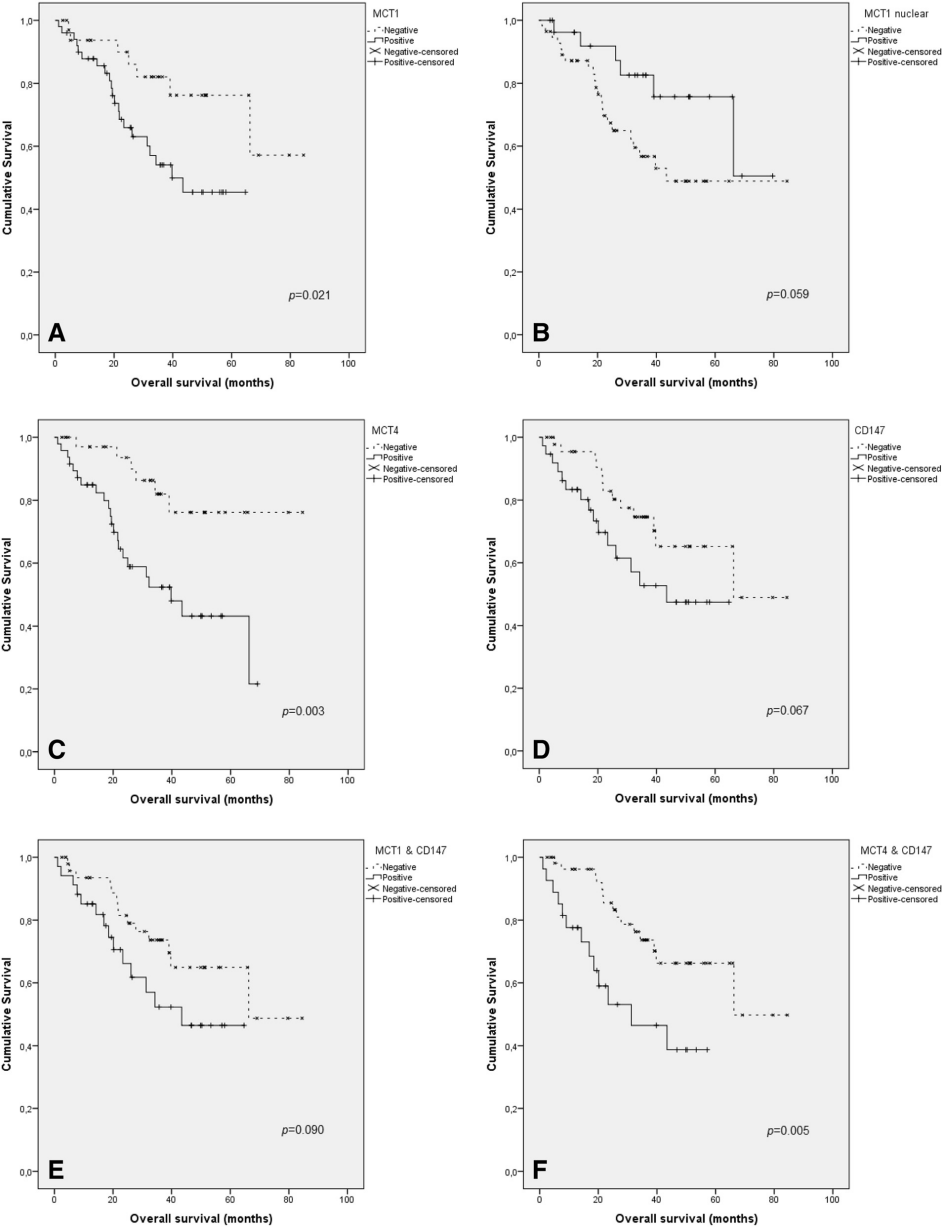
**Overall survival curves of soft tissue sarcomas’ patients.** The results are stratified according to the immunohistochemical expression of the different proteins analyzed. Continuous line refers to positive expression while interrupted line refers to negative expression. Plasma membrane expressions of MCT1 **(A)** and MCT4 **(C)** are significantly associated with lower patient’s overall survival, while nuclear expression of MCT1 shows a strong tendency to be associated with higher patient’s overall survival **(B)**. Plasma membrane expression of CD147 **(D)** is not associated with patient’s overall survival, however, plasma membrane co-expression of CD147 with MCT4 **(F)**, but not MCT1 **(E)**, is associated with lower patient’s overall survival.

Comparison of the survival rates between different expression phenotypes revealed an important association between the proteins analyzed in this study and the survival odds (Figure 2). Patients with either MCT1 or MCT4 plasma membrane positive tumors showed a significantly higher likelihood to present a lower overall survival than the negative cases; patients with MCT1 positive tumors showed a median overall survival of 25.5 months *versus* 34.2 months in the negative tumors (*p* = 0.021, Figure 2A), while patients with MCT4 positive tumors showed a median overall survival of 22.9 months *versus* 34.8 months in negative tumors (*p* = 0.003, Figure 2C). Importantly, MCT1 nuclear expression showed a border-line tendency to be associated with higher overall survival, with positive cases showing a median overall survival of 35.9 months *versus* 25.0 months in negative cases (*p* = 0.059, Figure 2B). A nearly significant result was also observed for CD147 (*p* = 0.067, Figure 2D), nevertheless, co-expression of CD147 along with MCT4 (Figure 2F), but not MCT1 (Figure 2E), showed a significant association with poorer overall survival, with patients with double MCT4/CD147 positive tumors showing a median overall survival of 19.5 months *versus* 34.6 months in the remaining group (*p* = 0.005).

### MCT1 nuclear expression is also found in an *in vitro* model of myxoid liposarcoma

**Figure 3:**
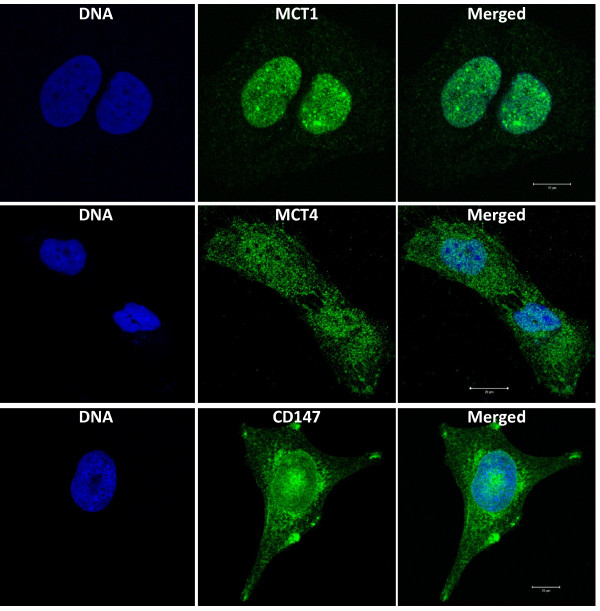
**Immunofluorescence for MCT1, MCT4 and CD147 in MLS-175 cells.** The myxoid liposarcoma cell line MLS-175 was used to evaluate the cellular localization of MCT1, MCT4 and CD147. MCT1 expression in the nucleus was confirmed, which was partly accompanied by CD147 expression. MCT4 was found homogeneously distributed through the cell. Images were obtained using a Zeiss LSM 510 Meta Confocal setup (Carl Zeiss, Oberkochen, Germany) equipped with a plan-Apochromat 100/1.4 oil objective.

**Figure 4:**
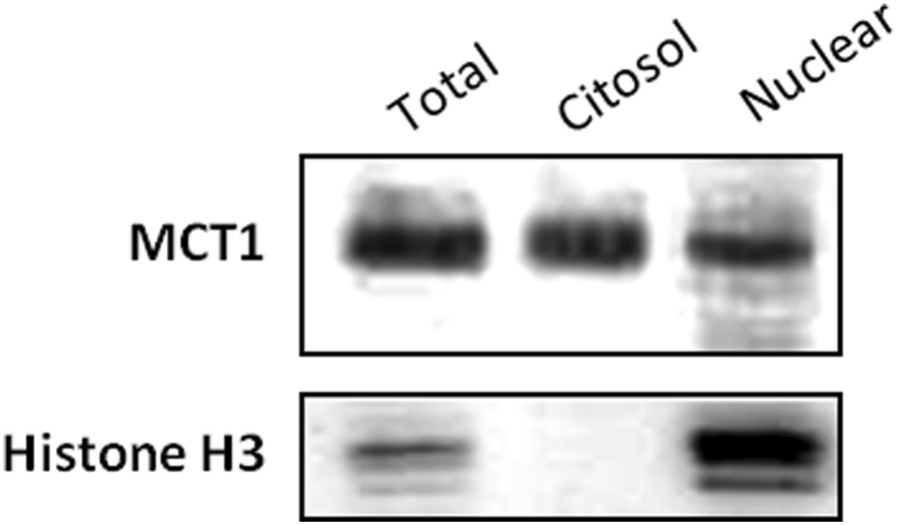
**Western blot of MCT1 after cell fractionation.** MLS-175 cells were used to extract nuclear and cytosolic proteins to evaluate the localization of MCT1. Images were obtained using the ChemiDoc XRS + system (BioRad).

To validate MCT1 expression in the nuclear compartment, immunofluorescence for MCT1, MCT4 and CD147 was performed in a sarcoma cell line representing the lineage with the highest frequency of MCT1 expression in the nucleus, the lipogenic lineage. As observed in Figure 3, MLS-1765 cells have a strong, and almost exclusive, expression of MCT1 in the nucleus. This MCT1 nuclear expression was partly accompanied by its chaperone CD147, which also seems to accumulate in the perinuclear region, while MCT4 was found homogeneously distributed through the cell. To further confirm the presence of MCT1 in the nucleus, we performed cell fractionation, separating the nuclear from the cytosolic fraction. As can be seen in Figure 4, the main protein band of MCT1 is present in the nuclear fraction, confirming the results of Figure 3.

## Discussion

Recent studies show that FDG-PET may contribute for precise grading and prognosis in different solid tumors, including soft tissue sarcomas [5], as high grade tumors show a much higher uptake of ^18^F-FDG due to a higher glycolytic phenotype [3]. Also, it was recently described that FDG-PET should be considered an important imaging modality for therapeutic monitoring in patients with high-grade STS [6,7]. In this context, metabolic characterization of STS emerges as a possibly relevant approach for STS management, with therapeutic implications as early treatment decisions such as discontinuation of chemotherapy in non-responding patients could be based on FDG-PET criteria [7]. Importantly, the hyperglycolytic phenotype present in this type of tumors, similarly to other solid tumors, may be the basis for the use of new directed therapeutic strategies which are currently in clinical trials [29]. Therefore, tumor metabolic characterization, including MCTs as responsible for lactate efflux from highly glycolytic cells, will have a relevant impact on predicting the group of patients that will benefit the most from this recent therapeutic approach and may have a prognostic value.

STSs are a very heterogeneous type of cancer, comprising over 50 histological subtypes, which are often associated with unique clinical, prognostic and therapeutic features [30]. Although some authors point to a lack of association between FDG uptake and histological type [4], others show that FDG uptake may differ significantly among histologic subtypes, when tumors of all grades were included [3]. Importantly, a low glycolytic phenotype was described in certain STS subtypes including tumors from the lipogenic lineage [3], which supports the low expression of MCT1 and MCT4 we found in the plasma membrane of this sarcoma subtype. This is in accordance with the low tumor cellularity characteristic of these tumors, which probably results in lower total tumor glucose consumption [3]. The low glycolytic rates shown by tumors from the lipogenic lineage could anticipate the expression of MCT2 in the plasma membrane in a way to allow cancer cells to obtain energy from oxidative phosphorylation, via uptake of other substrates like lactate itself or pyruvate; however, MCT2 was expressed in the cytoplasm of the majority of tumors, with only one tumor showing plasma membrane expression of this MCT isoform. Therefore, it seems that MCT2 lactate transport does not have a relevant role in sarcomas, as observed for other types of tumors [17,20]. When comparing tumor grade, MCT1, MCT4 and CD147 were able to distinguish between low and high grade sarcomas, reinforcing the description of a lower glycolytic phenotype in lower grade than in high grade sarcomas [3-5].

The molecular mechanisms underlying MCT expression in sarcomas were not under investigation in the present study, however, other studies have addressed this matter using the human rhabdomyosarcoma cell line RD [24,26]. Both MCT1 and MCT4 expressions were shown to be induced by the protein kinase C (PKC) signaling pathway [24,26], while MCT1 expression was shown to be inhibited by PKA signaling pathway [24]. However, additional studies are warranted to unveil the detailed downstream signaling pathway involved in MCT regulation, as well as other regulatory mechanisms.

It has been described that the hypoxia-induced emergence of a hyperglycolytic and acid-resistant phenotype will contribute to the invasiveness of cancer cells [31]. In this context, MCTs perform a dual role as lactate transporters, by extruding the end product of glycolysis, and pH regulators, by extruding a proton. Accordingly, in the present study, patients with disease progression have a higher likelihood to harbor tumors with plasma membrane expression of MCT1 and MCT4. Importantly, both MCT1 and MCT4 were able to distinguish between a group with worse survival (positive group) and a group with better survival (negative group), pointing to a possible value of MCTs in prognosis. This is the first study showing an association of MCTs with soft tissue sarcoma patient survival, which is in accordance with the role of these transporters in cancer as well as with their possible use as cancer therapeutic targets.

CD147, the MCT1 and MCT4 chaperone, is a much largely studied protein, due to its parallel function as a matrix metalloproteinase inducer [32]. In fact, CD147 expression has already been previously studied in a cohort of high grade soft tissue sarcomas, however, with no significant prognostic value [33]. In the present study, as the cohort also included low grade tumors, CD147 was significantly associated with high grade tumors. Importantly, when co-expressed with MCTs, CD147 was also significantly associated with other poor prognostic variables and patient lower survival, strengthening the hypothesis already raised in gastric cancer [34], that the prognostic value of CD147 is associated with its co-expression with MCTs, especially with MCT4 in the case of sarcomas. This suggests that the prognostic value of CD147 is mainly associated with its function as chaperone of MCTs, contributing in this way to the hyperglycolytic and acid-resistant phenotype.

Another major finding in the present study was the nuclear expression of MCT1, which was confirmed by immunofluorescence using a sarcoma cell line, and by cell fractionation, followed by Western blot. To the best of our knowledge, this is the first study showing nuclear expression of this protein. As the cellular localization does not fit with the classic role of this protein as a transmembrane transporter, this finding suggests the existence of an additional, not yet described, role of MCT1. Although interactions with other proteins are not well described for MCT1, some interactions can be found in the literature that can help to explain the mechanism underlying MCT1 expression in the nucleus. In fact, large-scale mapping of human protein-protein interactions by mass spectrometry shows an interaction of MCT1 with AP1S1 (clathrin-associated/assembly/adaptor protein, small 1) [35], which supports a mechanism of endocytosis (clathrin dependent endocytosis) that is shared by other proteins, like Notch1 and EGFR, that may alternatively lead to, instead of degradation, trafficking into the nucleus [36,37]. Therefore, similarly to other plasma membrane proteins which, upon a stimulus, can be directed to the nucleus and play additional roles as transcription modulators, we anticipate that MCT1 may be trafficked to the nucleus to perform an alternative function, not related to lactate transport activity. Other evidence on the factors governing MCT localization like the presence of specific sorting signals [38] or substrate-induced increase in MCT1 plasma membrane expression via GPR109A [39] may help to elucidate the mechanisms leading to MCT1 nuclear localization. Importantly, the presence of MCT1 in the nucleus appears to have a very different biological role than the one currently known, as tumors with nuclear expression of MCT1 show a completely opposite behavior when comparing to tumors expressing MCT1 in the plasma membrane; MCT1 nuclear expression was found to be associated with the lipogenic lineage, low grade tumors and higher overall survival, pointing to a probable role of this protein as tumor suppressor. Further studies are warranted to elucidate this possible new role of MCT1.

## Conclusions

This work gives an important contribution for the comprehension of the metabolic alterations occurring in STS, herein demonstrated to be dependent on the cell lineage, showing the value of MCT expression as a predictor of poor prognosis in these tumors. Importantly, the results herein presented provide the first evidence for a different type of MCT1 expression, which may change the current state of the art in what concerns monocarboxylate transporters, as it seems to be associated with a new biological role leading to a poor prognosis in STS.

## Abbreviations

MCTs: Monocarboxylate transporter; STSs: Soft tissue sarcomas; FDG-PET: ^18^F-fluorodeoxyglucose positron emission tomography.

## Competing interests

The authors declare that they have no competing interests.

## Authors’ contributions

CP carried out the immunohistochemical reactions (with FMS), performed the statistical analysis and drafted the manuscript. VP, MVO and ECC provided the clinical data of the cases. LM and SM performed the review of tumors’ diagnostic, histological grade and classification. GR carried out the immunohistochemical reactions. IV and DR performed the immunofluorescence. FMS performed the cell fractionation and Western blot. FCS evaluated the immunohistochemical reactions. RMR participated in the study design and coordination. FB conceived the study, participated in its design and coordination. All the authors read and approved the final manuscript.
